# Microstructures and Compressive Properties of Al Matrix Composites Reinforced with Bimodal Hybrid In-Situ Nano-/Micro-Sized TiC Particles

**DOI:** 10.3390/ma11081284

**Published:** 2018-07-25

**Authors:** Feng Qiu, Hao-Tian Tong, Yu-Yang Gao, Qian Zou, Bai-Xin Dong, Qiang Li, Jian-Ge Chu, Fang Chang, Shi-Li Shu, Qi-Chuan Jiang

**Affiliations:** 1 State Key Laboratory of Automotive Simulation and Control, Jilin University, Changchun 130025, China; qiufeng@jlu.edu.cn (F.Q.); tonght17@mails.jlu.edu.cn (H.-T.T.); gaoyy16@mails.jlu.edu.cn (Y.-Y.G.); dongbx1614@mails.jlu.edu.cn (B.-X.D.); liqiang17@mails.jlu.edu.cn (Q.L.); liuts9915@mails.jlu.edu.cn (J.-G.C.); changfang@jlu.edu.cn (F.C.); 2 Key Laboratory of Automobile Materials, Ministry of Education and Department of Materials Science and Engineering, Jilin University, Renmin Street NO. 5988, Changchun 130025, China; 3 Qingdao Automotive Research Institute of Jilin University, Qingdao 266000, China; 4 Department of Mechanical Engineering, Oakland University, Rochester, MI 48309, USA; qzou@oakland.edu; 5 State Key Laboratory of Luminescence and Applications, Changchun Institute of Optics, Fine Mechanics and Physics, Chinese Academy of Sciences, Changchun 130012, China; shushili@ciomp.ac.cn

**Keywords:** in-situ, bimodal -sized, combustion synthesis, TiC/Al

## Abstract

Bimodal hybrid in-situ nano-/micro-size TiC/Al composites were prepared with combustion synthesis of Al-Ti-C system and hot press consolidation. Attempt was made to obtain in-situ bimodal-size TiC particle reinforced dense Al matrix composites by using different carbon sources in the reaction process of hot pressing forming. Microstructure showed that the obtained composites exhibited reasonable bimodal-sized TiC distribution in the matrix and low porosity. With the increasing of the carbon nano tube (CNT) content from 0 to 100 wt. %, the average size of the TiC particles decreases and the compressive strength of the composite increase; while the fracture strain increases first and then decreases. The compressive properties of the bimodal-sized TiC/Al composites, especially the bimodal-sized composite synthesized by Al-Ti-C with 50 wt. % CNTs as carbon source, were improved compared with the composites reinforced with single sized TiC. The strengthening mechanism of the in-situ bimodal-sized particle reinforced aluminum matrix composites was revealed.

## 1. Introduction

Recently, the development of aluminum matrix composites (AMCs), which were reinforced by ceramic particles, has attracted considerable interests in many fields, such as aerospace, defense, and automotive applications [1,2,3]. These AMCs exhibited excellent mechanical properties, such as high specific strength, high wear resistance [4], low density, and low cost. However, most research work in this field has focused on the fabrication of the AMCs by ex-situ methods, such as conventional melting and casting, hot pressing, powder metallurgy technique, pressurized or un-pressurized infiltration, and so on [3,5,6,7,8,9]. In recent years, selective laser melting technology has also received more attention. It is a new type of metal powder rapid prototyping technology that can directly produce metal parts that are close to full density and good mechanical properties [10,11,12]. As is known, the ex-situ AMCs reinforced with ceramic particles have some problems, such as poor wettability, interface reactions, inhomogeneous distribution of reinforcement, etc., which result in the inferior mechanical properties [13,14,15]. On the contrary, the in-situ particle reinforced AMCs (PAMCs) provide finer particles, cleaner interfaces, and more homogeneous distribution comparatively of the particles. Recently, a new in-situ method for preparing particle reinforced composite material, i.e., combustion synthesis combined with hot press consolidation, was used to fabricate TiC/Al composites [16,17]. In this method, the reinforcing particles are directly generated inside the matrix by an exothermic reaction between elements. It possesses the obvious advantages, such as more uniform dispersion and finer sizes of ceramic particles, and stronger interfacial adhesion between reinforcement and matrix. TiC stands out among numerous reinforcement ceramic particles, which is mainly due to its good thermodynamic stability and wettability with molten Al, high melting point, ultra-high hardness, low coefficient of thermal expansion, and excellent wear resistance, the more important advantages is that it can be formed in-situ. As is known, the particle size of the reinforcement has great influence on the properties of PAMCs [18,19]. For a certain composite, the number of the particles increases as the particle size reduces, which has significant effect on the mechanical properties. Researchers have demonstrated that composites that were reinforced by nano-sized particle possess better properties, such as higher strength, higher hardness, outstanding ductility, and fracture toughness [18,20]. However, nano-particles might cause poor interface bonding and non-homogeneous dispersion, which has seriously hampered the strengthening effect, and affected the properties of the composites [21,22,23]. Also, more research work must be carried out to solve the problem of agglomeration of nano-particles dispersion. The micro-sized particles are more easily dispersed in Al matrix to improve the strength of the material [24]. Therefore, the bimodal-sized PAMCs provide a good idea on researching in light alloys a suitable balance of strength and ductility. Zhang et al. reported a research on the SiCp/Al2014 composites that were prepared by semi-solid stirring [25]. They found that the addition of 1 vol. % nano-SiCp particles enhanced the tensile strength from 503 to 585 MPa, and increased the fracture strain from 4.9% to 9.9%, when compared to the 4 vol. % micro-SiCp/Al2014 composites. By contrast with the extruded single-sized SiCp/Al2014 composites, the ultimate tensile and yield strength of bimodal-sized SiCp/Al2014 composites were remarkably enhanced. There were hardly any reports about the in-situ bimodal-sized (nano/micro) particle reinforced AMCs up to now, and the investigation on the effect of different ratios of two sized ceramic particles on the compressive behaviors of TiC/Al composites has not been reported either. In addition, the strengthening mechanism of the in-situ bimodal-sized particle reinforced aluminum matrix composites was studied simply in the present. 

In our previous work, nano-sized TiC particles of 40–100 nm were prepared by using carbon nano tube (CNTs) with the finer size and higher chemical activity as the carbon source [26,27], and micro-sized TiC particles were fabricated by using C black as carbon source [16,28]. When CNTs and C-black were used, the synthesized TiCx with particles sizes of 0.08–1 and 0.9–5 μm, respectively, was spherical or nearly spherical in shape. Based on these previous works, nano-/micro TiC/Al composites were prepared by using different ratios of CNTs and C black content. The microstructures and mechanical properties of in-situ bimodal-sized TiC/Al composites and the effect of the different ratios of the two sized ceramic particles on the compressive behaviors of TiC/Al composites have been investigated. The remarkable improvement of mechanical properties of the in-situ bimodal-sized TiC reinforced AMCs was obtained in our work. The optimal compressive properties, especially the fracture strain, of bimodal-sized TiC particle reinforced composite are higher than that of single-sized TiC particle reinforced composites. The research findings in this work will provide good guidance for the investigation and fabrication of in-situ bimodal-sized particle reinforced metal matrix composites. 

## 2. Materials and Methods

Commercial powders of Al (99.5% purity, ~28 μm), Ti (99.0% purity, ~28 μm), CNTs (diameter of 10 to 15 nm and length of 30 μm approximately), and C black (99.0% purity) were employed in the research. The carbon sources in the Al-Ti-C system were 0, 25, 50, 75 and 100 wt. % CNTs to fabricate 30 vol. % TiC in the composites, while the mass ratio of Ti/C (CNTs) was 1:1. The nominal compositions of Al-Ti-C systems were presented in Table 1. 

**Figure 1:**
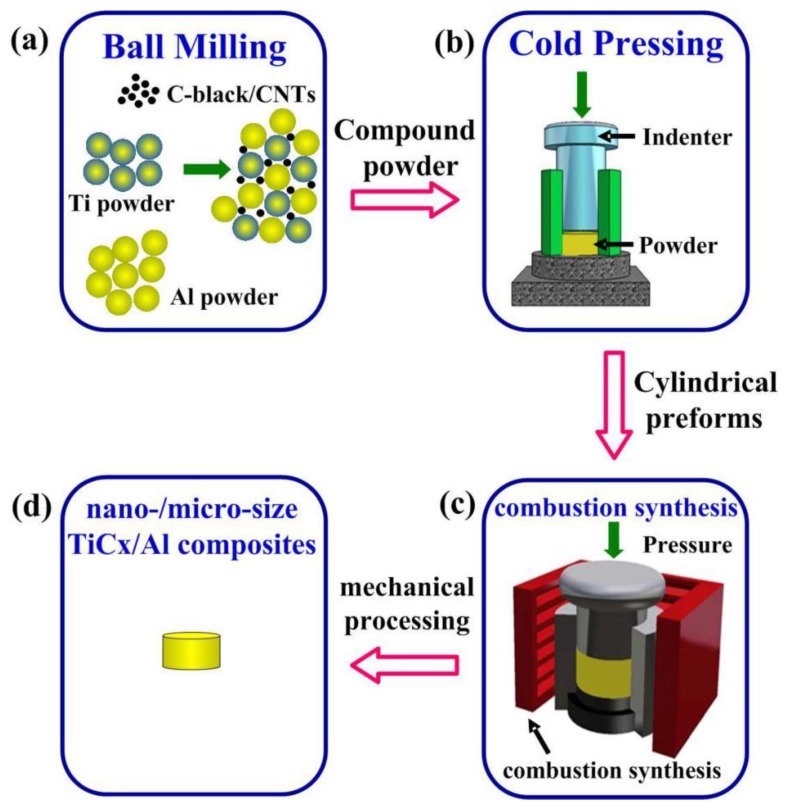
The process flow chart of nano-/micro-size TiC/Al composites.

**Figure 2:**
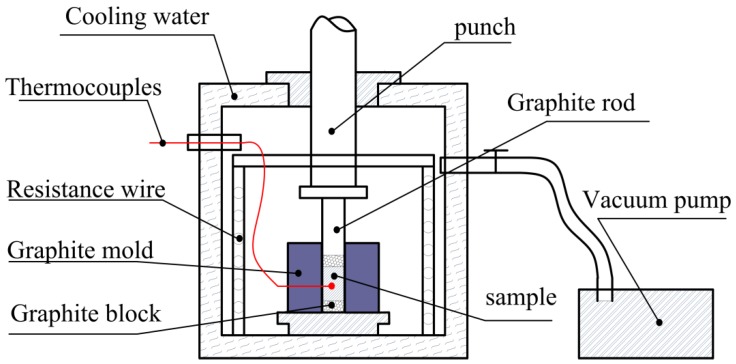
Detailed schematic of the equipment for the combustion synthesis and hot press consolidation.

Figure 1 shows the process flow chart of nano-/micro-size TiC/Al composites. The materials were mixed sufficiently with low-speed ball milling (24 h, 50 rpm approximately) and cold pressed into cylindrical samples (diameter of 45 mm and length of 35 mm) afterwards. After the milling, the real oxygen contents in mixed powder were analyzed by the pulse heating inert gas fusion–infrared thermal conductivity method, and the oxygen impurity is 0.2–0.3 at. %. The cylindrical sample was put into a vacuum thermal explosion furnace (Shenyang jinyan new material preparation technology Co. Ltd., Shenyang, Liaoning, China), Detailed schematic is shown in Figure 2) with the heating rate of about 30 K/min and the temperatures were measured by W5-Re26 thermocouples (Shenyang Dongda Sensor Technology Co. Ltd., Shenyang, Liaoning, China). The sudden rapid rise in temperature was the sign of that the sample had been ignited. The sample was quickly pressed on while its temperature is still high and then cooled down to the room temperature. A small amount of the mixed Al-Ti-C powder was performed for differential thermal analysis (DTA, TA SDT-Q600, New Castle, DE, USA) experiment to research the effects of different carbon sources on the reaction process of Al-Ti-C system. The sample was heated to 1273 K in an alumina crucible with a heating rate of 30 K/min.

The X-ray diffraction (XRD, Moldel D/Max 2500PC, Tokyo, Japan) with Cu Kα radiation was used to study the phase constituent of the materials. The SEM (Model Evo18, Carl Zeiss, Oberkochen, Germany) and Field Emission Scanning Electron Microscope (FESEM, JSM 6700F, Tokyo, Japan) were applied in order to investigate the microstructure of the Al matrix and the size distribution of the particles. In compression experiments, the cylindrical samples (diameter of 3 mm and height of 6 mm) were tested. The uniaxial compression test was carried out with a strain rate of 1 × 10^−4^ s^−1^ by a servo-hydraulic materials testing system (MTS, MTS 810, Minneapolis, MN, USA). The fracture surface of the products was observed by SEM. The average particle size of the nano-/micro-sized TiC was measured by a Nano Measurer (self-developed measurer software). 

## 3. Results and Discussion

**Figure 3:**
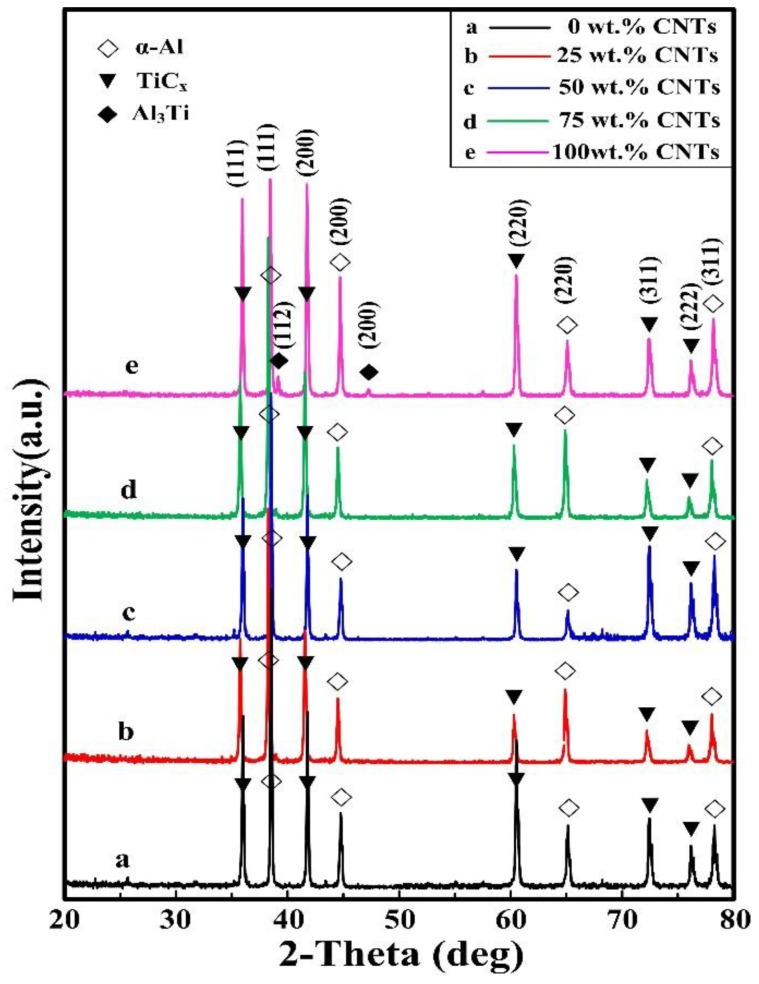
The X-ray diffraction (XRD) patterns of the 30 vol. % TiC/Al composites synthesized by Al-Ti-C system with different carbon nano tube (CNT) contents as the carbon source.

Figure 3 shows the XRD patterns of the 30 vol. % TiC/Al composites synthesized by Al-Ti-C with different CNT contents as the carbon source. It shows that the final products mainly include α-Al and TiC. In addition, a small amount of Al3Ti intermetallic compound was inspected in the composite that was synthesized by Al-Ti-C with 100 wt. % CNTs as the carbon source. It indicated that, when the CNTs content in the Al-Ti-C system was high, the reaction of the system tended to be incomplete.

**Figure 4:**
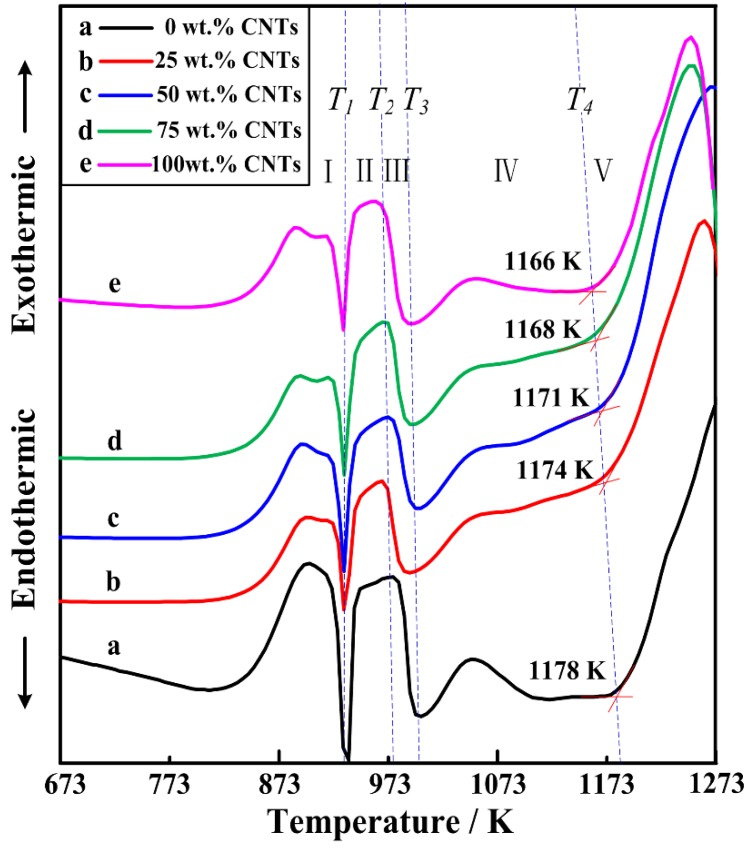
The differential thermal analysis curves of the 30 vol. % TiC/Al composites synthesized by Al-Ti-C system with different CNT contents as the carbon source.

On behalf of developing a better comprehend of reaction process in the Al-Ti-C/CNTs systems, DTA experiments were employed in the Al-Ti-C/CNTs mixtures. Figure 4 is the DTA curves collection for the Al-Ti-C powders with different CNT contents as the carbon source. These DTA curves show similar thermodynamic behaviors, which indicated the influence of carbon sources on the reaction process of the Al-Ti-C system. On the basis of the results, the mechanism of the reaction is a model of reaction-dissolution-precipitation. With the increase of the temperature, aluminum melted at temperature T_1_, subsequently, Al_3_Ti was first generated in the Al-Ti-C system and then melted to form a binary Al-Ti liquid phase at temperature T_2_. Simultaneously, the binary Al-Ti liquid phase spread over the C, subsequently led to the formation of ternary Al-Ti-C liquid phase at temperature T3. When the concentration of C and Ti were high enough, TiC was generated and precipitated out of the melts. An obvious exothermic peak started from T_3_, and continued to ~1073 K. It indicated that the formation of ternary Al-Ti-C liquid phase and a small quantity of TiC promoted the exothermic event. With the increase of the temperature, it indicated that the exothermic event at temperature T_4_ was caused by the formation of a large number of TiC. With the increase of the content of CNTs in the Al-Ti-C system, the TiC generating temperature (T4) decreased from 1178 to 1166 K.

**Figure 5:**
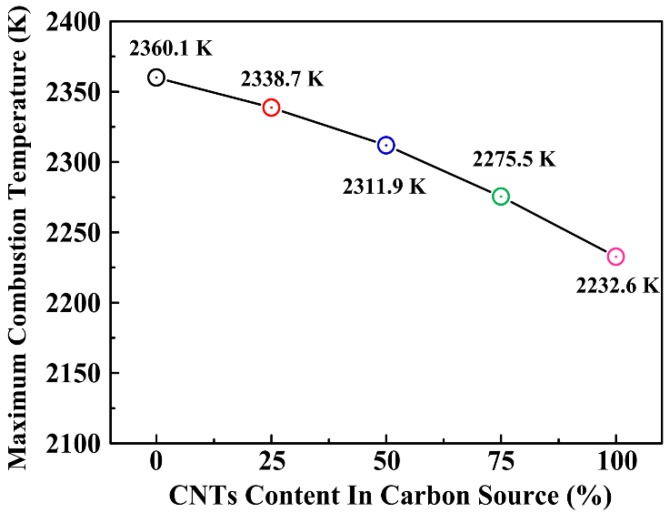
Variation of the maximum combustion temperature in the Al-Ti-C system with different CNT contents as the carbon source.

This phenomenon is due to the acceleration of the dissolution when more CNTs as the carbon source were used. Because of the increase of the CNTs, the area of contact between the Al-Ti liquid phase and CNTs extends, which promotes the reaction, and the reaction starts at a lower initial temperature. Figure 5 shows the variation of the maximum combustion temperature with different CNT content as the carbon source. It was shown that the maximum combustion temperature decreased rapidly from 2360.1 to 2232.6 K, with the increase of the CNT contents from 0 to 100 wt. %. When the CNT content is too high, e.g., 100 wt. %, the maximum combustion temperature of the reaction system will be reduced greatly, then the combustion synthesis reaction will behindered and incomplete, and Al3Ti will be detected. In a word, the more CNTs are used, the lower temperature is measured, which may affect the reaction process in the system.

**Figure 6:**
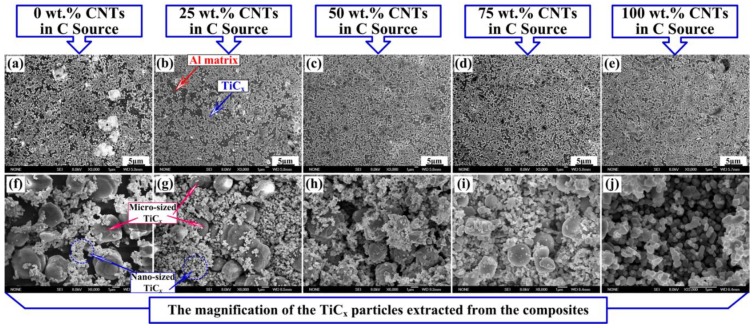
The Field Emission SEM (FESEM) images of the 30 vol. % TiC/Al composites synthesized by Al-Ti-C system with different CNT contents as the carbon source, (**a**) 0, (**b**) 25, (**c**) 50, (**d**) 75, and (**e**) 100 wt. %, respectively, figures (**f**–**j**) are the magnification of the TiC particles extracted from the composites corresponding to the figures (**a**–**e**), respectively.

**Figure 7:**
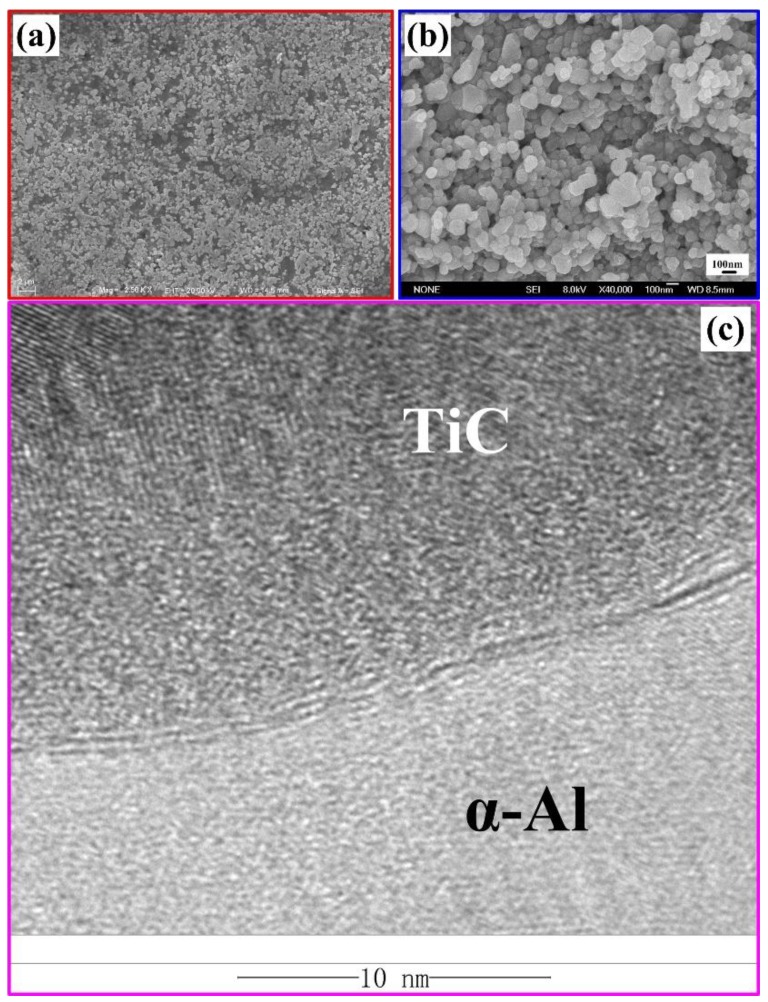
(**a**) The SEM images of the 30 vol. % TiC/Al composites synthesized by Al-Ti-C system with 50 wt. % CNT as the carbon source, (**b**) the magnification of the TiC particles extracted from the composites synthesized by Al-Ti-C system with 100 wt. % CNT as the carbon source, and (**c**) High Resolution Transmission Electron Microscope image of interface between α-Al and nano-sized TiC in 30 vol. % TiC/Al composites synthesized by Al-Ti-C system with 50 wt. % CNT as the carbon source.

Figure 6 shows the FESEM images of the 30 vol. % TiC/Al composites that were synthesized by Al-Ti-C system with different CNT contents as the carbon source. In Figure 6b, the microstructure consists of two different zones: the bright white phase is corresponding to TiC and the rest is corresponding to the Al matrix. It shows that the obtained composites exhibit reasonable TiC distribution in the matrix with low porosity. With CNT contents changing from 0 to 100 wt. %. The composites synthesized in Al-Ti-C systems with carbon source of mixed C-black/CNTs showed the most homogenous distribution of nano-sized TiC. Figure 7a shows the SEM images of the 30 vol. % TiC/Al composites synthesized by Al-Ti-C system with 50 wt. % CNT as the carbon source. It can be seen that the ceramic particles with brighter colors distribute evenly in the aluminum matrix, the deeper color area is aluminum matrix with lower porosity. Figure 6f–i are the magnification of the TiC particles that were abstracted from the Al matrix using 18 vol. % HCl-distilled water solution. Table 2 lists the average size of the micron-sized and nano-sized TiC particles. It exhibits that the in-situ bimodal-sized (nano and micro) TiC/Al composites are fabricated, and the average particle size of both nano/micro-sized TiC and the volume fraction of micro-sized TiC decrease when CNT contents changing from 0 to 100 wt. %. The average particle sizes of nano-sized and micro-sized TiC decrease from 265.3 nm and 3.44 μm to 175.2 nm and 1.25 μm, respectively, and the micro-sized TiC does not appear in the TiC/Al composite synthesized with 100 wt. % CNTs as the carbon source. The volume ratio of the micro-sized to nano-sized particles decreases from 59.19% to 0 with CNTs changing from 0 to 100 wt. %. Figure 7b shows the FESEM magnification of the TiC particles extracted from the composites synthesized by Al-Ti-C system with 100 wt. % CNT as the carbon source. The size of TiC nanoparticles is clearly visible, and the average particle size is 175.25 nm. For the TiC/Al composite that was synthesized with 50 wt. % CNTs as the carbon source, the average particle sizes of the micro-sized and nano-sized TiC are about 1.94 μm and 240.86 nm, respectively. 

**Figure 8:**
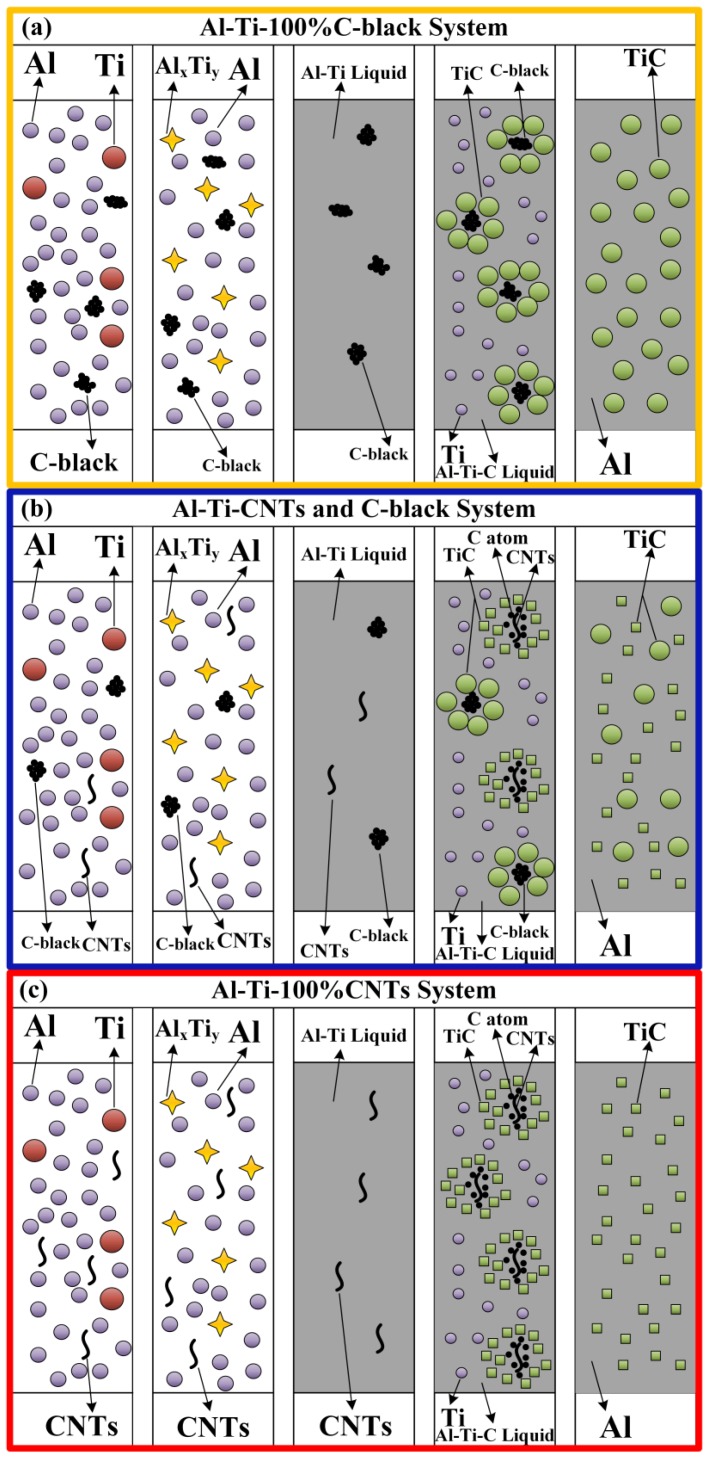
Schematic diagram showing the above-mentioned formation process of TiC particles in the Al-Ti-C system with different CNT contents as the carbon source: (**a**) Al-Ti-100%C-black system, (**b**) Al-Ti-CNTs and C-black system, and (**c**) Al-Ti-100%CNTs system.

Figure 8 is a schematic diagram showing the above-mentioned formation process of TiC particles in the Al-Ti-C system with different CNT contents as the carbon source, and details of the whole process can be understood intuitively from the figure. During the reaction process, when compared with finer-sized carbon source, more heat will be needed when large-sized carbon source dissolves, which finally causes the increase in the combustion temperature [29]. With CNT contents changing from 0 to 100 wt. %, the decrease in combustion temperature leads to a reduction in the size of TiC particles. On the other hand, the apparent reactivity of the Al-Ti binary liquid phase with the carbon source is an important influencing factor on the reaction process. In comparison with the C black, CNTs have finer size, and the defects, such as vacancies, heptagons, and pentagons in the structure of the CNTs, endow it with more chemical activity [30]. Thus, TiC particles synthesized with CNTs as the carbon source have the smallest size. At the same time, the synthesized TiC particles with smaller size tend to be octahedral in shape due to the fact that the dissolution of carbon is limited at low combustion temperatures [27,31,32]. The apparent reactivity of the binary Al-Ti liquid phase with the CNTs seems to be higher than that with the C-black [29]. Hence, the TiC particles that were synthesized by using C-black with spherical shape have the largest size. The synthesized TiC particles with large size tend to be spherical or near-spherical in shape at high combustion temperatures. 

**Figure 9:**
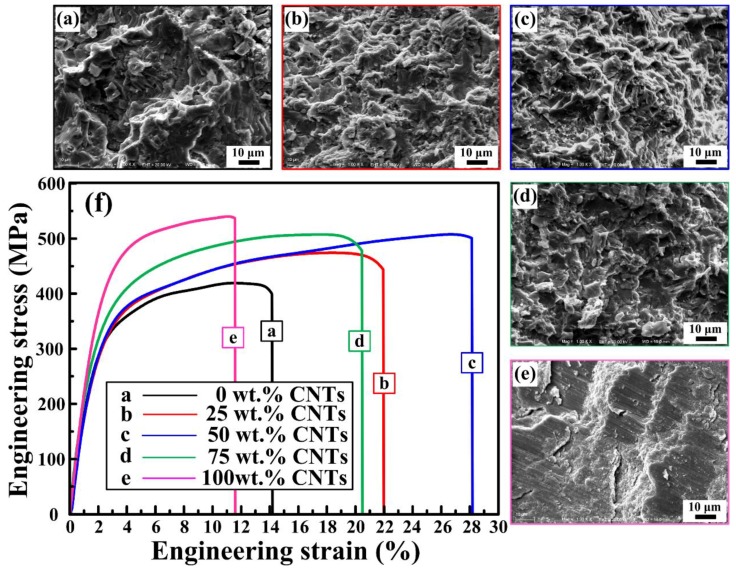
(**a**–**e**) SEM images of the compression fracture surfaces of the 30 vol. % TiC/Al composites synthesized by Al-Ti-C system with different CNT contents as the carbon source, (**a**) 0, (**b**) 25, (**c**) 50, (**d**) 75, and (**e**) 100 wt. %, respectively. (**f**) Compressive stress-strain curves of the 30 vol. % TiC/Al composites synthesized by Al-Ti-C system with different CNT contents as the carbon source.

**Figure 10:**
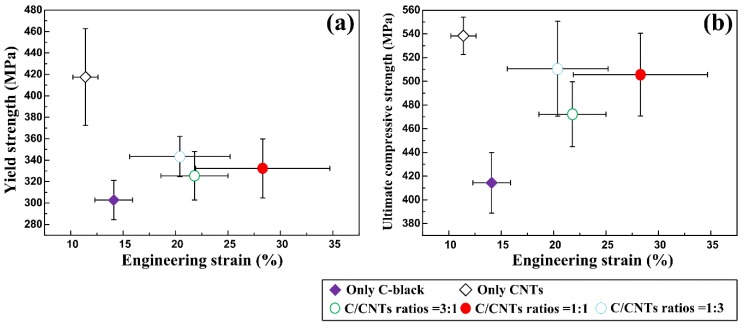
The comparisons in (**a**) compressive yield strength vs. engineering strain and (**b**) ultimate compressive strength vs. engineering strain of the 30 vol. % TiC/Al composites that were synthesized by Al-Ti-C with different CNT contents as the carbon source.

Figure 9 are the SEM images of the compression fracture surfaces and compressive stress-strain curves of the 30 vol. % TiC/Al composites that were synthesized by Al-Ti-C system with different CNT contents as the carbon source. Figure 9f shows compressive stress-strain curves of them. The compressive properties of the composites are enumerated in Table 3. Figure 10 is the comparisons in compressive yield strength vs. engineering strain and ultimate compressive strength vs. engineering strain of them. On the elastic part of the compression curve, the difference in elastic modulus is not obvious, and the compression curves of the elastic part are basically overlapping, with some minor differences. The reason for this phenomenon is that as the content of CNTs increases, the number of nano-ceramic particles increases greatly, the size decreases remarkably, resulting in a large increase in the interfaces between the ceramic particles and the matrix, and the interface bonding is excellent, as shown in Figure 7c, therefore, the modulus of elasticity has increased slightly. The yield strength (σ_0.2_), ultimate compressive strength (σ_UCS_), and fracture strain (ε_f_) of TiC/Al composites are enhanced with the CNT contents changing from 0 to 50 wt. % in the system. With the CNT contents changing from 50 to 100 wt. %, the σ_0.2_ and σ_UCS_ of TiC/Al composites further increase, while εf reduces gradually. Among all of the composites, the one synthesized by Al-Ti-C with 100 wt. % CNTs as the carbon source possesses the σ_0.2_ and σ_UCS_ of 417.5 MPa and 538.3 MPa, while the ε_f_ of 11.4%, which is the lowest one. The composite synthesized by Al-Ti-C with 50 wt. % CNTs is optimized to have the best comprehensive properties: the σ_UCS_ of 505.6 MPa and the ε_f_ of 28.3%. Al-Ti-C with different CNT contents as the carbon source. 

Figure 9a–e are the SEM images of the compression fracture surfaces of the composites synthesized by Al-Ti-C with different CNT contents. The composite synthesized by Al-Ti-C with 50 wt. % CNTs as the carbon source shows the highest fracture strain and the corresponding fracture surface is covered with near-equiaxed dimples. On the contrary, the lack of dimples in Figure 9f gives evidence to the appearance of brittle fracture. It also indicates that the composite synthesized by Al-Ti-C with 100 wt. % CNTs as the carbon source shows the lowest ε_f_ due to the existence of some brittle intermediate phase Al_3_Ti. During the hot-pressing process, the concentration of dislocations may occur around TiC, which leads to stress concentration at the interface between TiC and α-Al phases. More micro-sized TiC in composites would intensify the stress concentration. While more nano-sized TiC in composites would benefit from stress dispersion in the matrix. The stress exceeding the interfacial bond strength might lead the generation of microcracks at the interface between TiC and α-Al phases. Internal microcracks may cause the fracture of the composite. As shown in Figure 7c, clean micron-sized particle/matrix interfacial bonding without micropores or cracks could be observed. The size decreases remarkably, resulting in a large increase in the interfaces between the ceramic particles and the matrix, and the interface bonding is excellent. A large number of dispersed nanoparticles, and the good interfaces between nanoparticles and α-Al would help to reduce stress concentration and improve toughness or plasticity of aluminum alloy. In Tian’s works, a small amount of hybrid-size TiCp/Al-Cu composites contain 1.0 wt. % micro-sized TiC and 0.3 wt. % nano-sized TiC possessed the highest fracture strain of 17.5%, improved by 133.3% when compared with the Al-Cu matrix alloy. They believe that a strong bonding interface is expected to improve the overall properties of composites [33]. Therefore, the optimal compressive properties, especially the fracture strain, of bimodal-sized TiC particle reinforced Al matrix composite is higher than that of the single-sized TiC particle reinforced composites. 

The presence of the nano-sized TiC in the aluminum resulted in remarkably improvement in the compressive strength. The yield strength and compressive strength are mainly controlled by nano-sized TiC, owing to their pinning effect on hindering the movement of the dislocations and grain boundaries. Moreover, the micro-sized TiC resulted in the strain hardening in the area near the particles; the mismatch of the coefficient of thermal expansion and the elastic modulus between the aluminum matrix and the ceramic particles may cause the dislocations during the solidification and hot press. The micro-sized TiC content increases, the strain hardening effect increases approximately, and because of the Orowan mechanism, the dislocation glide is hindered by nano-sized particles. Besides, the strong interfacial bonding between the particles and the Al matrix was beneficial for effective load transfer from the Al matrix to the particles, thus improving the strength of the composites. Therefore, the strengthening mechanism of composites that are reinforced with bimodal hybrid in-situ TiC particles is mainly due to the dislocation density strengthening mechanism, Orowan strengthening mechanism, load bearing effect, and strong interface strengthening. 

## 4. Conclusions

The in-situ bimodal-sized (nano-/micro) TiC/Al composites were successfully obtained using a different ratio of CNT and C contents as the carbon sources in the Al-Ti-C system. With CNT contents changing from 0 to 100 wt. %. The composites that were synthesized in Al-Ti-C systems with carbon source of mixed C-black/CNTs showed more homogenous distribution of nano-sized TiC. With the CNT contents changing from 0 to 100 wt. %, the volume fraction of the micro-sized TiC decrease, and the average particle sizes of nano-sized and micro-sized TiC decrease from 265.3 nm and 3.44 μm to 175.2 nm and 1.25 μm, respectively, because of the decrease of the combustion temperature. The σ_0.2_, σ_UCS_, and ε_f_ of TiC/Al composites are dramatically enhanced with the CNT contents changing from 0 to 50 wt. %. With the CNT contents changing from 50 to 100 wt. %, the σ_0.2_ and σ_UCS_ of TiC/Al composites further increase, while ε_f_ reduces gradually. The composite synthesized with 50 wt. % CNTs as the carbon source is optimized to have the best comprehensive properties: the σ_UCS_ of 505.6 MPa and the ε_f_ of 28.3%. It is found that the optimal compressive properties, especially the fracture strain, of bimodal-sized TiC particle reinforced composites are higher than those of single-sized TiC particle reinforced composites. The introduction of the nano-sized TiC effectively controls its α-Al grain growth. Thus, the yield strength and compressive strength are mainly controlled by nano-sized TiC, owing to their pinning effect on hindering the movement of the dislocations and grain boundaries. Moreover, the micro-sized TiC resulted in the strain hardening in the area near the particles; the mismatch of the coefficient of thermal expansion and the elastic modulus between the aluminum matrix and the ceramic particles may cause the dislocations during the solidification and hot press. The strengthening mechanism of composites that are reinforced with bimodal hybrid in-situ TiC particles is mainly due to the dislocation density strengthening mechanism, Orowan strengthening mechanism, load bearing effect, and strong interface strengthening. 

## Figures and Tables

**Table 1:** Characteristics of the in situ TiC/Al composites synthesized in Al-Ti-C systems.

Samples	Composition of Mixed Raw Material Powders
30 vol. % TiC/Al (0 wt. % CNTs)	70 wt. % Al + 24 wt. % Ti + (6.0 wt. % C black + 0.0 wt. % CNTs)
30 vol. % TiC/Al (25 wt. % CNTs)	70 wt. % Al + 24 wt. % Ti + (4.5 wt. % C black + 1.5 wt. % CNTs)
30 vol. % TiC/Al (50 wt. % CNTs)	70 wt. % Al + 24 wt. % Ti + (3.0 wt. % C black + 3.0 wt. % CNTs)
30 vol. % TiC/Al (75 wt. % CNTs)	70 wt. % Al + 24 wt. % Ti + (1.5 wt. % C black + 4.5 wt. % CNTs)
30 vol. % TiC/Al (100 wt. % CNTs)	70 wt. % Al + 24 wt. % Ti + (0.0 wt. % C black + 6.0 wt. % CNTs)

**Table 2:** The average size and the volume ratios of the TiC particles.

C/CNTs Ratios	Micro-Size of TiC (μm)	Nano-Size of TiC (nm)	Volume Ratios (%)
C	3.44	265.31	59.19
3:1	2.43	250.08	44.72
1:1	1.94	240.86	37.14
1:3	1.25	233.04	5.362
CNTs	---	175.25	0.0

**Table 3:** Densities and compressive properties of the 30 vol. % TiC/Al composites synthesized by Al-Ti-C with different CNT contents as the carbon source.

C/CNTs Ratios	σ0.2 (MPa)	σUCS (MPa)	εf (%)
C	302.8 ± 18.4	414.4 ± 25.6	14.1 ± 1.8
3:1	325.4 ± 22.5	472.2 ± 27.4	21.8 ± 3.2
1:1	332.3 ± 27.6	505.6 ± 34.8	28.3 ± 6.4
1:3	343.2 ± 18.8	510.5 ± 40.0	20.4 ± 4.8
CNTs	417.5 ± 45.2	538.3 ± 15.8	11.4 ± 1.2
